# The Burden of Illness Related to Chronic Obstructive Pulmonary Disease Exacerbations in Québec, Canada

**DOI:** 10.1155/2017/8184915

**Published:** 2017-06-20

**Authors:** Tam Dang-Tan, Shiyuan Zhang, Ruben V. Tavares, Melissa Stutz, Afisi S. Ismaila, Julie Vaillancourt, Diane Corriveau, Richard H. Stanford, Xiwu Lin, Gilbert A. Nadeau, Alexander Simidchiev, Daria Parsons, John S. Sampalis

**Affiliations:** ^1^GlaxoSmithKline, Research Triangle Park, Durham, NC, USA; ^2^GlaxoSmithKline, Mississauga, ON, Canada; ^3^JSS Medical Research Inc., Saint Laurent, QC, Canada; ^4^Department of Clinical Epidemiology and Biostatistics, McMaster University, Hamilton, ON, USA; ^5^GlaxoSmithKline, Collegeville, PA, USA; ^6^McGill University, Montreal, QC, Canada

## Abstract

**Background:**

Chronic obstructive pulmonary disease (COPD) prevalence in Canada has risen over time. COPD-related exacerbations contribute to the increased health care utilization (HCU) in this population. This study investigated the impact of exacerbations on COPD-related HCU.

**Methods:**

This retrospective observational cohort study used patient data from the Québec provincial health insurance databases. Eligible patients with a new HCU claim with a diagnostic billing for COPD during 2001–2010 were followed until March 31, 2011. Exacerbation rates and time to first exacerbation were assessed. Unadjusted analyses and multivariable models compared the rate of HCU by exacerbation classification (any [moderate/severe], moderate, or severe).

**Results:**

The exacerbation event rate in patients with an exacerbation was 34.3 events/100 patient-years (22.7 for moderate exacerbations and 11.6 for severe exacerbations). Median time to first exacerbation of any classification was 37 months. In unadjusted analyses, COPD-related HCU significantly increased with exacerbation severity. In the multivariable, HCU rates were significantly higher after exacerbation versus before exacerbation (*p* < 0.01) for patients with an exacerbation or moderate exacerbations. For severe exacerbations, general practitioner, respiratory specialist, emergency room, and hospital visits were significantly higher after exacerbation versus before exacerbation (*p* < 0.001).

**Conclusions:**

Exacerbations were associated with increased HCU, which was more pronounced for patients with severe exacerbations. Interventions to reduce the risk of exacerbations in patients with COPD may reduce disease burden.

## 1. Introduction

Chronic obstructive pulmonary disease (COPD) is a progressive disease with self-reported diagnoses in 4% of Canadians aged 35–79 years [1]; as many as 17% of Canadians have a lung function score indicative of COPD [2]. COPD is the leading cause of hospitalizations for ambulatory care conditions in Canada [3], with an estimated annual direct health care cost of more than CAD 1.5 billion during the past decade [4]. Despite a reduction in COPD incidence and all-cause mortality over the past decade in Canada, the prevalence of COPD has increased [5].

The impact of COPD-related exacerbations on burden of illness has been assessed in several studies [4, 6–9]. COPD exacerbations significantly influence patient lung function and quality of life and are associated with increased morbidity and mortality [10]. The average incidence of exacerbations in patients with moderate to severe COPD is estimated at approximately 0.6–3.0 per year, depending on the population, region, duration of follow-up, and definition of exacerbation used [11–17].

Existing studies on COPD-related HCU are limited. Given the chronic nature of COPD, patients can be treated by several physicians and be covered by more than one insurance plan. This can lead to loss of information regarding HCU. These issues limit the generalization of the results to the general COPD population. In addition, in observational prospective studies, the total cost of COPD and related exacerbations may be underestimated because of limited follow-up time and recall bias. Larger population-based studies with a long-term follow-up and comprehensive ascertainment of HCU are required to provide a complete assessment of the burden of illness related to COPD exacerbations. This longitudinal study aimed to investigate the impact of COPD exacerbations on disease-related HCU in Québec, Canada.

## 2. Methods

### 2.1. Study Objectives

The primary objective was to describe the burden of illness related to COPD exacerbations in Québec, Canada. Burden of illness was determined based on the incidence of COPD-related exacerbations and HCU related to the management of COPD exacerbations. Secondary objectives were to describe the profile of COPD exacerbations in terms of severity, frequency, characteristics (e.g., medication use, hospitalizations, and emergency room [ER] visits), and time to first exacerbation and to estimate the risk of a COPD exacerbation based on patient characteristics, disease parameters, and treatments.

### 2.2. Study Design

**Figure 1:**
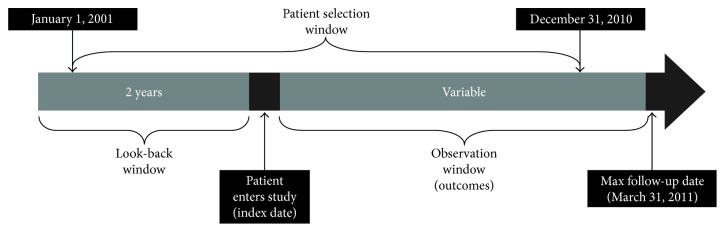
Data extraction time frame.

This was a retrospective observational cohort study of patients with COPD using deidentified patient data derived from provincial health insurance databases (Régie de l'Assurance Maladie du Québec [RAMQ]). Eligible patients classified with COPD were selected from a random sample of 250,000 with a health administrative claim linked with an International Classification of Diseases, Ninth Revision (ICD-9) diagnostic billing for either asthma or COPD. Patients were followed from the date of COPD classification (index date), until March 31, 2011, death, or withdrawal from the health insurance plan. A 2-year look-back or run-in period prior to the index date was included to assess eligibility and detect comorbidities (Figure 1). The burden of comorbidities was calculated using the Charlson Comorbidity Index (CCI), adapted for use with the RAMQ databases. In addition, the presence of coronary artery disease, congestive heart failure, hypertension, secondary pulmonary hypertension, ventricular dysfunction, ischemic heart disease, cardiac failure, diabetes, obstructive sleep apnea, cancer, osteoporosis, diabetes metabolic syndrome, depression, and normocytic anemia was used to describe the comorbidity profile of patients with COPD [18, 19].

A moderate exacerbation was defined as a physician visit with a diagnosis code for COPD and an oral corticosteroid (OCS) or an antibiotic prescription for a respiratory infection (filled within 2 weeks of the physician visit), whereas a severe exacerbation was defined as an ER visit with a primary diagnosis code for COPD or hospitalization with a primary discharge diagnosis code for COPD. To ascertain the study outcomes, all RAMQ medical and pharmaceutical claims and Med-Écho hospitalization records for the patients included in the study were extracted from the index date to March 31, 2011. Survival status was determined by record linkage by the Institut de la Statistique de Québec Death Registry.

The study was conducted in accordance with the Declaration of Helsinki. Access to the data, for the purpose of this study, was granted by the Commission d'Accès à l'Information du Québec, the data custodian. At the time of the data acquisition, external research ethics board approval was not required.

### 2.3. Patient Eligibility

Eligible patients ≥ 40 years of age at treatment initiation had a new HCU claim with a diagnostic billing for COPD between January 01, 2001, and December 31, 2010, with a respiratory medicine prescription claim within 2 weeks of the diagnostic billing claim for COPD. To assess eligibility and detect comorbidities, patients were also required to have available data in the RAMQ database for the 2-year look-back period preceding the index date. Eligible HCU claims for disease classification included physician visit or ER visits or hospitalization. The COPD diagnostic billing included either chronic bronchitis or emphysema (ICD-9 codes: 490.xx, 491.xx, 492.xx, or 496.xx). Respiratory medicines considered for eligibility included are shown in Box 1. Patients were excluded from the study if they had a COPD HCU claim (physician or hospitalization) or prescription during the 2-year look-back period. Additionally, patients with a medical or prescription claim for asthma (ICD-9: 493.xx), respiratory tract cancer (160.xx–164.xx or 231.xx), cystic fibrosis (277.xx), fibrosis due to tuberculosis (TB) (011.4; 011.40–0.11-46) and bronchiectasis (494.xx), pneumoconiosis (505.xx), pulmonary fibrosis (515.xx), pulmonary TB (011.xx), or sarcoidosis (135.xx) at any time during the look-back or follow-up periods were also excluded.

### 2.4. Study Outcomes

The primary study outcome was the number of exacerbation-related HCU events per year of follow-up. Annual COPD exacerbation rates during the follow-up period and time to first COPD exacerbation since diagnosis of COPD were assessed as secondary study outcomes. Study outcomes were assessed according to whether they experienced no or at least one exacerbation (any moderate or severe event), a moderate exacerbation (but no severe exacerbations) only, or severe exacerbation (patients in the “severe” category may have also had a moderate exacerbation) during the follow-up period. Diary assessment data were not available for the assessment of mild exacerbation events. Utilization of the following health care resources was assessed: prescriptions for rescue medications (e.g., short-acting *β*_2_ agonists [SABAs]), maintenance medications (e.g., long-acting *β*_2_ agonists [LABAs], long-acting muscarinic agonists [limited to tiotropium during study period], inhaled corticosteroids [ICS], and ICS/LABA combinations), antibiotics, or OCS; COPD-related ER visits, hospitalizations, and intensive care unit (ICU) admissions, including length of stay and intubations; and COPD-related visits to general practitioners (GPs) and respiratory specialists.

### 2.5. Statistical Analyses

Descriptive statistics were produced for all relevant study variables including patient characteristics, disease parameters, and treatments. Unadjusted analyses and multivariable Poisson regression models were used to compare patient groups according to exacerbation classification with respect to the rate of HCU. In unadjusted analyses, between-group differences in HCU were analyzed using incidence density rate ratios (IDRRs). For the multivariable models, the covariables included age, gender, baseline CCI score, baseline comorbidity profile, follow-up duration, and HCU in the look-back period; follow-up duration was used as an offset variable. The time to first exacerbation was described using the Kaplan-Meier estimate of the survival function as measured from the time of diagnosis of COPD.

## 3. Results

### 3.1. Study Population

A total of 53,349 patients with COPD met the study eligibility criteria and were included, of whom 25,259 (47.3%) experienced no (“no”) COPD exacerbations and 28,090 (52.7%) experienced at least one COPD exacerbation during the follow-up period. The majority of patients were female (57.6%) and the mean (SD) age was 68.1 (12.8) years at the index date. The total study population was followed for a mean (standard deviation [SD]) of 4.43 (2.70) years (Table 1). At baseline, SABAs, antibiotics, and OCS were dispensed to 13.5%, 36.3%, and 11.5% of patients, respectively.

### 3.2. Burden of Disease

#### 3.2.1. Exacerbation Rates and HCU during the Follow-Up Period

The exacerbation rate during the follow-up period was 34.3 events per 100 patient-years in patients experiencing an exacerbation; 22.7 events per 100 patient-years in patients experiencing only moderate exacerbation; and 11.6 events per 100 patient-years in patients experiencing at least one severe exacerbation. Of the patients who experienced an exacerbation, 17,288 (61.5%) experienced moderate events, while 10,802 (38.5%) experienced at least one severe exacerbation during the follow-up period. Compared with patients who experienced “no” or only moderate exacerbations, patients who experienced severe exacerbations were older (mean [SD] age of 75.1 [9.9] years), were mostly male (54.5%), and had a higher use of medications and more medical visits during the follow-up period (Table 1).

#### 3.2.2. Time to First COPD Exacerbation

**Figure 2:**
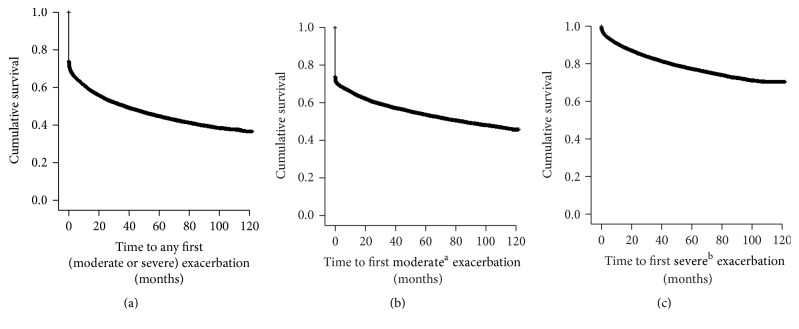
Kaplan-Meier plot showing time to first COPD exacerbation for (a) an exacerbation, (b) moderate exacerbation, and (c) severe exacerbation. COPD, chronic obstructive pulmonary disease. ^a^A moderate exacerbation was defined as a physician visit with a diagnosis code for COPD and an OCS or an antibiotic prescription for a respiratory infection (filled within 2 weeks of the physician visit). Patients included experienced ≥1 moderate exacerbation but no severe exacerbations. ^b^A severe exacerbation was defined as an ER visit with a primary diagnosis code for COPD or hospitalization with a primary discharge diagnosis code for COPD. Patients included experienced ≥1 severe exacerbation but may also have ≥1 moderate exacerbation.

Median time to a first COPD exacerbation was 37 months from the index date (Figure 2(a)). Median time to a first moderate exacerbation was 85 months (Figure 2(b)). Fewer than 50% of patients experienced a severe COPD exacerbation (Figure 2(c)).

### 3.3. Relative Risk of HCU

#### 3.3.1. Unadjusted Analyses

**Figure 3:**
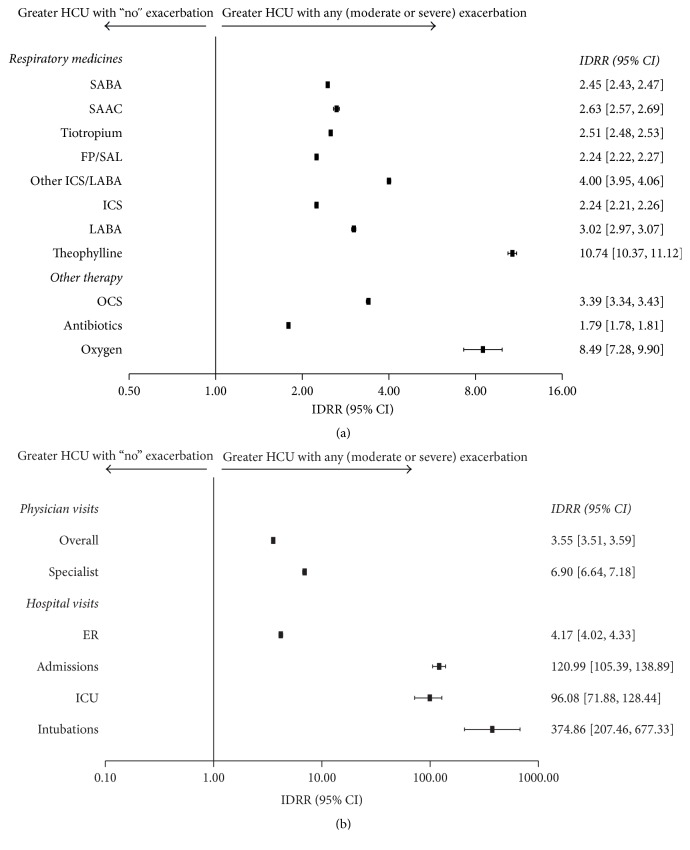
Relative risk of health care resource utilization for (a) COPD therapy and (b) health care visits in patients experiencing ≥1 exacerbation (*N* = 17,288) versus no exacerbations (*N* = 25,259) (IDRR). CI, confidence interval; COPD, chronic obstructive pulmonary disease; ER, emergency room; FP/SAL, fluticasone propionate/salmeterol; HCU, health care resource utilization; ICS, inhaled corticosteroid; ICU, intensive care unit; IDRR, incidence density rate ratio; LABA, long-acting *β*_2_ agonist; OCS, oral corticosteroid; SAAC, short-acting anticholinergic; SABA, short-acting *β*_2_ agonist.

**Figure 4:**
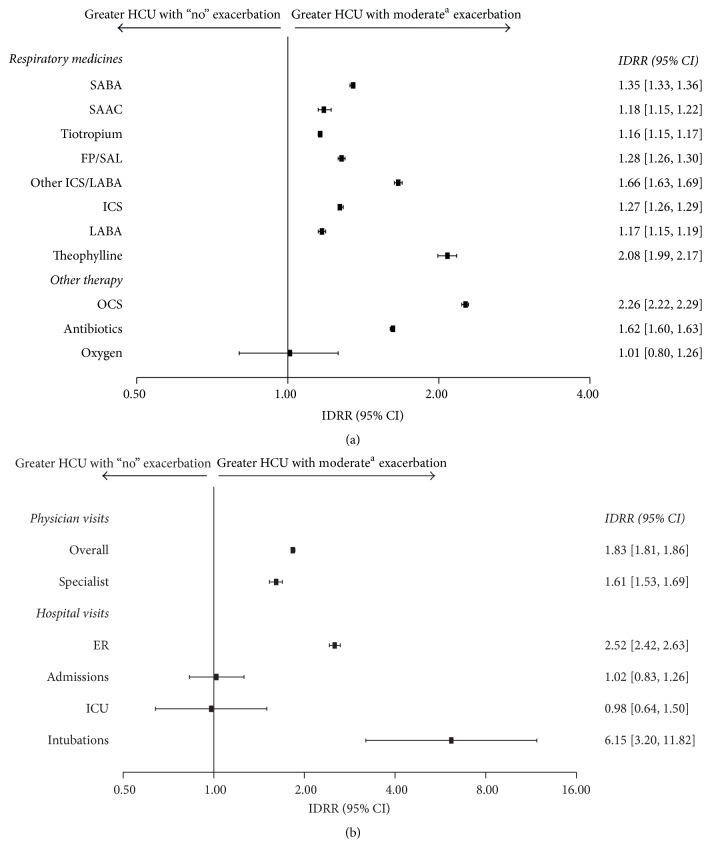
Relative risk of health care resource utilization for (a) COPD therapy and (b) health care visits in patients experiencing moderate exacerbations (*N* = 28,090) versus no exacerbations (*N* = 25,259) (IDRR). ^a^A moderate exacerbation was defined as a physician visit with a diagnosis code for COPD and an OCS or an antibiotic prescription for a respiratory infection (filled within 2 weeks of the physician visit). Patients included in this column experienced ≥1 moderate exacerbations but no severe exacerbations; CI, confidence interval; COPD, chronic obstructive pulmonary disease; ER, emergency room; FP/SAL, fluticasone propionate/salmeterol; HCU, health care utilization; ICS, inhaled corticosteroid; ICU, intensive care unit; IDRR, incidence density rate ratio; LABA, long-acting *β*_2_ agonist; OCS, oral corticosteroid; SAAC, short-acting anticholinergic; SABA, short-acting *β*_2_ agonist.

**Figure 5:**
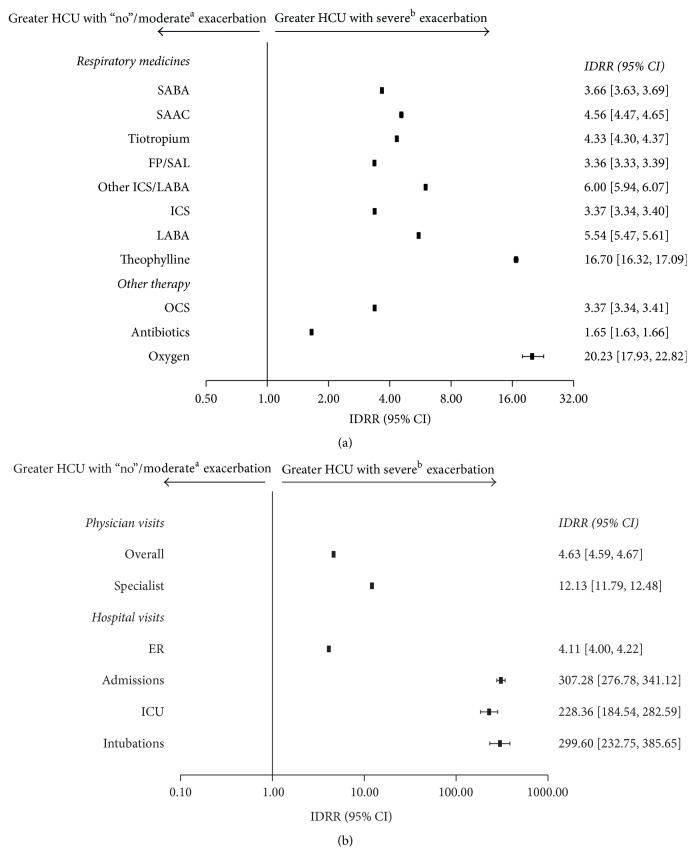
Relative risk of health care resource utilization for (a) COPD therapy and (b) health care visits in patients experiencing ≥1 severe exacerbation (*N* = 10,802) versus “no” or ≥1 moderate exacerbation (*N* = 42,547) (IDRR). ^a^A moderate exacerbation was defined as a physician visit with a diagnosis code for COPD and an OCS or an antibiotic prescription for a respiratory infection (filled within 2 weeks of the physician visit). Patients included in this column experienced ≥1 moderate exacerbation but no severe exacerbations. ^b^A severe exacerbation was defined as an ER visit with a primary diagnosis code for COPD or hospitalization with a primary discharge diagnosis code for COPD; patients included in this column experienced ≥1 severe exacerbation but may also have ≥1 moderate exacerbation; CI, confidence interval; COPD, chronic obstructive pulmonary disease; ER, emergency room; FP/SAL, fluticasone propionate/salmeterol; HCU, health care resource utilization; ICS, inhaled corticosteroid; ICU, intensive care unit; IDRR, incidence density rate ratio; LABA, long-acting *β*_2_ agonist; OCS, oral corticosteroid; SAAC, short-acting anticholinergic; SABA, short-acting *β*_2_ agonist.

Patients who experienced a COPD exacerbation had significantly higher HCU rates across all parameters compared with patients who experienced “no” exacerbations during the follow-up period (Figure 3; *p* < 0.001). Patients experiencing only moderate exacerbations had significantly higher HCU rates across most parameters compared with patients who experienced “no” exacerbations during the follow-up period (Figure 4; *p* < 0.001). Patients who experienced severe exacerbations had significantly higher HCU rates across all parameters compared with patients who experienced only moderate or “no” exacerbations during the follow-up period (Figure 5; *p* < 0.001).

#### 3.3.2. Multivariable Analyses

Adjustment for demographics, comorbidities, and prior HCU did not directionally alter the results. As with the unadjusted analyses, in patients who experienced an exacerbation or moderate exacerbation, HCU was higher during the 3 months after an exacerbation compared with preexacerbation HCU (Table S1 in Supplementary Material available online at https://doi.org/10.1155/2017/8184915; *p* < 0.01). Similarly, GP, respiratory specialist, ER, and hospital visits were significantly increased during the 3 months after a severe exacerbation compared with the number of preexacerbation visits (*p* < 0.001), although the use of antibiotics and OCS were not significantly different before versus after exacerbation (Table S1).

## 4. Discussion

In this retrospective observational cohort study of patients with COPD using the Québec, Canada, provincial health insurance databases, patients experiencing exacerbations had a significantly higher HCU rate compared with those patients who did not experience exacerbations. The effect was more pronounced in patients who experienced severe exacerbations. These results are consistent with those reported previously [4, 7, 9, 20–22]. For example, a retrospective claims-based analysis conducted among commercially insured patients in the USA showed that the average direct annual health cost for the management of COPD was $2,003 to $43,461 (2006 USD) per patient depending on the type of patient and care received [21]. Additionally, the Resource Utilization Study in COPD (RUSIC), a longitudinal study that followed 609 patients with COPD recruited from 50 sites across Canada for 52 weeks, documented a high prevalence of COPD exacerbations that impacted HCU [4, 22]. The study found that the average health cost for a moderate exacerbation was approximately $756 and that for a severe exacerbation was $9,953. The cost drivers for moderate exacerbations were ER visits and mode of transportation to the ER, and for severe exacerbations, the cost drivers were length of hospital stay and inpatient laboratory and diagnostic testing. Extrapolating these results to the Canadian population, the estimated total cost of COPD exacerbations in Canada was $646 million to $736 million [4]. Similarly, studies in Europe have also shown that exacerbation-related health costs increase with COPD exacerbation severity and that the higher costs in patients with severe exacerbations are driven by higher HCU of hospitalizations, physician visits, and prescriptions for treatments [7, 9, 20]. However, the Salford Lung Study demonstrated that fluticasone furoate/vilanterol reduces the frequency of moderate or severe exacerbations compared with usual COPD standard of care but interestingly was not associated with a reduction in HCU [23]. This may suggest HCU is driven by factors other than exacerbation severity.

However, direct comparisons between studies are challenging due to differences in methodology, quality of evidence, and regional standards of care. Recently, in the Salford Lung Study, a randomized clinical trial designed to reflect the reality of clinical practice, comparative groups were based on allocated treatment rather than exacerbation severity as in the current study, which may have resulted in treatment groups with mixed exacerbation frequencies. The Salford Lung Study also had a shorter duration of patient follow-up (1 year) compared with the current study (average 4.44 years), providing a shorter period of time for HCU assessment and also enrolled patients prospectively, potentially biasing the patient inclusion away from the general COPD population [23]. A moderate exacerbation was defined as a physician visit with a diagnosis code for COPD and OCS or an antibiotic prescription for a respiratory infection (filled within 2 weeks of the physician visit), whereas a severe exacerbation was defined as an ER visit with a primary diagnosis code for COPD or hospitalization with a primary discharge diagnosis code for COPD. The definitions used in the Swedish study by Andersson et al. [20] were similar to those used in our study; a moderate exacerbation was defined as requiring a GP visit or outpatient facility, whereas a severe exacerbation required an ER visit or hospital admission. In comparison, RUSIC defined a moderate exacerbation as a visit to an outpatient facility, including ER visits not requiring hospitalization, and an alteration in medication and severe exacerbations as those requiring hospitalization [4, 22]. Oostenbrink and Rutten-van Mölken [7] defined a moderate exacerbation as causing discomfort enough to cause interference with usual activity and a severe exacerbation as incapacitating or causing inability to do work or usual activity. Despite their differences in exacerbation definition, the results of our study and of those previously reported support the notion that exacerbations are the main drivers of the high cost of the management of COPD.

Limitations of this study include those that are typical of administrative database studies. The most important one of these limitations is the validity of the information included in the database, specifically relating to the diagnosis and treatment of COPD, which were based on ICD codes and claims submissions. Indeed, a previous study suggested that many RAMQ diagnoses of COPD lacked validity [24]. In our study, in addition to the ICD codes used for COPD diagnosis, the requirement for treatment with respiratory-specific medications and the exclusion of diagnoses in the differential reduced the likelihood of misdiagnoses and were similar to the criteria applied in a previous database study [25]. Another potential limitation is that our study only considered moderate and severe COPD exacerbations. We did not have access to diary information to assess mild exacerbations, which are usually defined as a sustained increase in symptoms that does not lead to the prescription of additional treatment (antibiotics or ICS) or to a hospitalization. Unreported symptom-based events are frequent and have a similar negative impact on health status to reported events, suggesting these mild events are of clinical interest [26]. Finally, we need to consider the generalizability of provincial health insurance administrative databases to the overall COPD population. However, the RAMQ datasets contain administrative data from Québec residents covered by the provincial health care and public drug insurance plans. There are approximately 7.9 million individuals covered by Québec provincial health care insurance and approximately 44% of the total population of the province of Québec (3.5 million individual subscribers) is covered by the public drug insurance plan [27]. The public drug insurance plan covers individuals without any private insurance, welfare recipients, and individuals aged ≥ 65 years. The RAMQ Pharmaceutical Services database, which contains data on the claims reimbursed by the Québec public drug insurance plan, has been validated for use in pharmacoepidemiologic research and is regarded as one of the most well-characterized drug dispensation datasets covering individuals in Québec [28]. These databases have been used extensively in pharmacoepidemiologic studies [25, 29–31]. The RAMQ database provides a reasonable sample of patients with COPD in Québec. Because Québec has the second largest population in Canada, estimates from this population may be used to make inferences to burden of illness at the national level.

## 5. Conclusions

This retrospective observational cohort study confirmed that patients with COPD and exacerbations contribute significantly to the COPD-related burden of illness. We found that COPD exacerbations were associated with increased HCU in patients with COPD in Québec, Canada. This effect was more pronounced for patients who experienced severe exacerbations compared with no or moderate exacerbations. The study findings support that reducing the risk of exacerbations in patients with COPD could substantially reduce the health care burden of COPD in Québec, Canada.

## Supplementary Material

Following adjustment for demographics, comorbidities and prior HCU, in patients who experienced an exacerbation or moderate exacerbation, HCU was higher during the 3 months after an exacerbation compared with pre-exacerbation HCU

## Figures and Tables

**Box 1:**
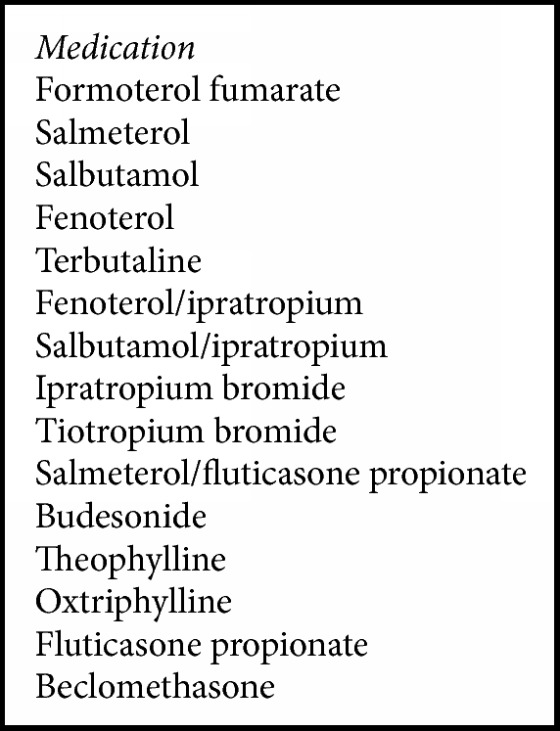
COPD medication prescriptions for determining patient eligibility.

**Table 1:** Patient demographics and baseline characteristics.

	Exacerbation classification				Total study population *N* = 53,349
	“No” *N* = 25,259	Any (moderate^a^ or severe)^b^ *N* = 28,090	Moderate^a^ *N* = 17,288	Severe^b^ *N* = 10,802	
*Follow-up, years *					
Sum	107,206	129,529	79,218	50,310	236,735
Mean (SD)	4.24 (2.66)	4.61 (2.70)	4.73 (2.70)	4.66 (2.73)	4.43 (2.70)
*Age, years*					
Mean (SD)	65.85 (13.12)	70.10 (12.21)	66.96 (12.48)	75.13 (9.87)	68.09 (12.83)
*Gender, female*					
*n* (%)	60.0	55.5	61.8	45.5	57.6
*Comorbidity* ^*c*^ * (%) *					
Respiratory disease^d^	14.5	26.9	34.2	45.6	21.0
Hypertension	6.2	11.2	13.5	20.0	8.9
Depression	4.2	7.8	9.4	13.7	6.1
Diabetes	6.7	11.9	12.7	22.2	9.4
*CCI score*					
mean (SD)	1.00 (1.67)	1.40 (1.87)	0.92 (1.58)	2.15 (2.03)	1.21 (1.79)
*Prior medications (prescriptions/patient)* ^*e*^					
*Antibiotics*					
Mean (SD)	0.77 (2.20)	0.83 (2.40)	0.80 (2.17)	0.88 (2.71)	0.80 (2.31)
*OCS*					
Mean (SD)	0.28 (1.94)	0.62 (4.49)	0.49 (4.15)	0.83 (4.98)	0.46 (3.52)
*SABA *					
Mean (SD)	0.41 (2.29)	0.86 (3.74)	0.41 (2.63)	1.58 (4.95)	0.64 (3.15)
*LABA/ICS*					
Mean (SD)	0.07 (1.25)	0.16 (1.74)	0.08 (1.30)	0.30 (2.28)	0.12 (1.53)
*Prior medical visits (visits/patient; COPD-related)* ^*e*^					
*GP*					
Mean (SD)	0.40 (0.94)	0.58 (1.64)	0.25 (0.90)	1.10 (2.29)	0.50 (1.35)
*Specialist*					
Mean (SD)	0.06 (0.43)	0.11 (0.68)	0.03 (0.34)	0.23 (0.99)	0.08 (0.57)
*ER*					
Mean (SD)	0.10 (0.33)	0.10 (0.38)	0.05 (0.25)	0.19 (0.51)	0.10 (0.36)
*Hospitalization*					
Mean (SD)	0.06 (0.25)	0.18 (0.42)	0.04 (0.21)	0.39 (0.55)	0.12 (0.35)

^a^A moderate exacerbation was defined as a physician visit with a diagnosis code for COPD and an OCS or an antibiotic prescription for a respiratory infection (filled within 2 weeks of the physician visit). Patients included in this column experienced ≥1 moderate exacerbations but no severe exacerbations. ^b^A severe exacerbation was defined as an ER visit with a primary diagnosis code for COPD or hospitalization with a primary discharge diagnosis code for COPD; patients included in this column experienced ≥1 severe exacerbation but may also have ≥1 moderate exacerbations; ^c^concomitant conditions prevalent in at least 5% of the total population reported; ^d^prevalent COPD and other concomitant respiratory conditions at baseline per eligibility criteria; ^e^during the 2-year look-back window. CCI, Charlson Comorbidity Index; COPD, chronic obstructive pulmonary disorder; ER, emergency room; GP, general practitioner; ICS, inhaled corticosteroids; LABA, long-acting *β*_2_ agonist; OCS, oral corticosteroids; SABA, short-acting *β*_2_ agonist; SD, standard deviation.
