# Supplementary material for: The Burden of Illness Related to Chronic Obstructive Pulmonary Disease Exacerbations in Québec, Canada
Source: Can Respir J. 2017 Jun 20;2017:8184915. doi: 10.1155/2017/8184915 (PMC5496115; doi:10.1155/2017/8184915)
Supplement: Supplementary file 1 — Following adjustment for demographics, comorbidities and prior HCU, in patients who experienced an exacerbation or moderate exacerbation, HCU was higher during the 3 months after an exacerbation compared with pre-exacerbation HCU [file 8184915.f1.doc]

**Table S1.** Change in patient HCU pre-exacerbation compared with the 3-month post-exacerbation

|  | **Exacerbation classification** | | |
| --- | --- | --- | --- |
|  | **Any**  **(moderate^a^ or severe)^b^**  **N=27,756** | **Moderate^a^**  **N=21,822** | **Severe^b^**  **N=10,802** |
| **Change in patient HCU pre-exacerbation compared with the 3-month post-exacerbation** | | | |
| **Antibiotics** mean change in use (95% CI)  Events/patient | 0.41 (0.40, 0.42)  0.91 | 0.75 (0.74, 0.76)  1.18 | -0.01 (-0.03, 0.00)  -0.04 |
| **OCS**  mean change in use (95% CI)  Events/patient | 0.19 (0.18, 0.20)  0.43 | 0.36 (0.35, 0.37)  0.57 | -0.02 (-0.03, 0.004)  -0.04 |
| **GP** mean change in visits (95% CI)  Events/patient | 0.81 (0.78, 0.84)  1.80 | 0.86 (0.84, 0.88)  1.35 | 0.75 (0.69, 0.80)  1.89 |
| **Specialist**  mean change in visits (95% CI)  Events/patient | 0.21 (0.19, 0.23)  0.47 | 0.04 (0.02, 0.06)  0.06 | 0.43 (0.39, 0.46)  1.08 |
| **ER**  mean change in visits (95% CI)  Events/patient | 0.07 (0.07, 0.08)  0.16 | 0.18 (0.17, 0.18)  0.28 | -0.06 (-0.07, -0.05)  -0.15 |
| **Hospitalization**  mean change in visits (95% CI)  Events/patient | 0.40 (0.39, 0.40)  0.88 | 0.01 (0.00, 0.01)  0.01 | 0.89 (0.88, 0.90)  2.25 |

^a^A moderate exacerbation was defined as a physician visit with a diagnosis code for COPD and an OCS or an antibiotic prescription for a respiratory infection (filled within 2 weeks of the physician visit). Patients included in this column experienced ≥1 moderate exacerbations but no severe exacerbations. ^b^A severe exacerbation was defined as an ER visit with a primary diagnosis code for COPD, or hospitalization with a primary discharge diagnosis code for COPD; Patients included in this column experienced ≥1 severe exacerbation but may also have ≥1 moderate exacerbations;

CI, confidence interval; COPD, chronic obstructive pulmonary disease; ER, emergency room;
GP, general practitioner; HCU, health care utilization; OCS, oral corticosteroid
